# Mandibular Movement Restoration in a Child with Bilateral Coronoid Hyperplasia: A Case Report

**DOI:** 10.3889/oamjms.2016.049

**Published:** 2016-04-21

**Authors:** Danica Popovik Monevska, Alberto Benedetti, Vladimir Popovski, Slave Naumovski, Aleksandar Grcev, Suzana Bozovic, Aleksandar Stamatoski

**Affiliations:** 1 *University Clinic for Maxillofacial Surgery, Faculty of Dental Medicine, Ss Cyril and Methodius University of Skopje, Skopje, Republic of Macedonia*; 2 *Faculty of Dental Medicine, Ss Cyril and Methodius University of Skopje, Skopje, Republic of Macedonia*

**Keywords:** coronoid hyperplasia, resection, intraoral approach, physiotherapy, Temporomandibular joint disorders

## Abstract

**BACKGROUND:**

Coronoid process hyperplasia is an uncommon finding, characterized by an enlargement of the coronoid process, causing a mechanical obstacle by its interposing in the posterior portion of the maxilla or zygomatic arch.

**CASE PRESENTATION:**

The article presents a case report of a bilateral coronoid process hyperplasia in a 3-year-old girl demonstrated with inability to open the mouth and restricted jaw movement. Panoramic x-ray and 3-dimensional computed tomographic reconstruction showed bilateral elongation of the coronoid processes associated with deformation of the mandibular condyle with no involvement of the articular space. A coronoid resection by intraoral approach was done, followed by an aggressive physiotherapy. A considerable improvement in mouth opening of 30 mm was achieved. We strongly suggest early surgical treatment of coronoid hyperplasia to recover morphology and function consequently to reduce skeletofacial deformities in young patients.

**CONCLUSIONS:**

The article presents a clinical and surgical case of bilateral coronoidectomy in a 3-year-old girl, with retrognathic mandible. The diagnosis of bilateral coronoid process hyperplasia was confirmed, and the surgical treatment was under general anesthesia, with nasotracheal intubation guided by a nasofiber endoscope, using an intraoral approach.

## Introduction

The coronoid process hyperplasia is an uncommon finding, characterized by an enlargement of the coronoid process, causing a mechanical obstacle by interposition of this structure in the posterior portion of the maxilla or zygomatic arch. The most important feature of this condition is a mouth opening reduction [1, 2]. Its etiology and pathogenesis is still unknown although several theories have been proposed [3].

Among conventional radiographic image techniques, the oblique posteroanterior, modified Town’s for condyles and computed tomography with three-dimensional reconstructions are used for accurately evaluation of the coronoid process [4].

This case report is about a patient with bilateral mandibular coronoid process hyperplasia treated with coronoidectomy, followed with intensive physical therapy. To the best of our knowledge, this is the youngest patient so far reported with such bilateral anomaly.

## Clinical Case

**Figure 1:**
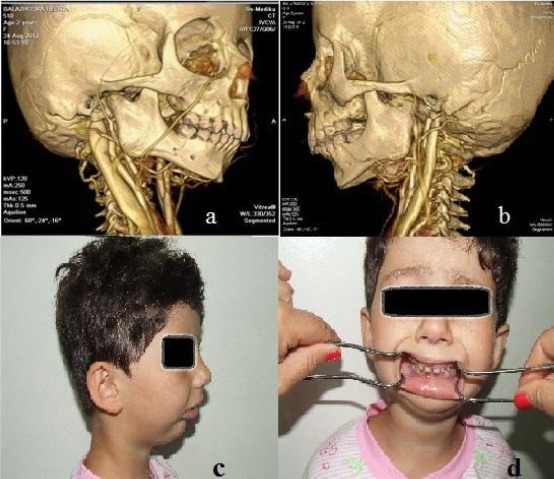
*Preoperative three-dimensional CT: a. Right lateral view: the enlargement of the coronoid process protruding under inferior rim of the zygomatic arch; b. Left lateral view: the enlargement of the coronoid process protruding under the inferior rim of the zygomatic arch; c.d. lateral and frontal preoperative photograph (mouth opening prior to operation)*.

A 3-year-old girl was referred to the Clinic for Maxillofacial Surgery with inability to open her mouth, restricted jaw movement and limited mastication. Convex profile, severely retrognathic mandible and absence of chin button were present. Intraoral examination revealed mouth opening of only 1-2 mm (Fig. 1).

Preoperative assessment included anamnesis, radiographic and physical investigation. Parents could not recollect the time of onset of mouth opening restriction. Child’s photos from the first months of life showed no restrictions of the mouth opening, indicating that the condition was not caused by any trauma during delivery.

Panoramic radiographs and 3-dimensional computed tomographic reconstruction confirmed bilateral elongation of the coronoid processes associated with deformation of the mandibular condyle with no involvement of articular space (Fig. 1). It appears from the CT scan that the coronoid hyperplasia is secondary to the TMJ ankylosis that is present in the left TMJ.

Using Bard-Parker blade, intraoral incisions (first right, than left) were made bilaterally along the buccal mucosa (anterior border - margo anterior of mandible) at the level of occlusal plane and extended posteriorly up to the anterior faucial pillars.

**Figure 2:**
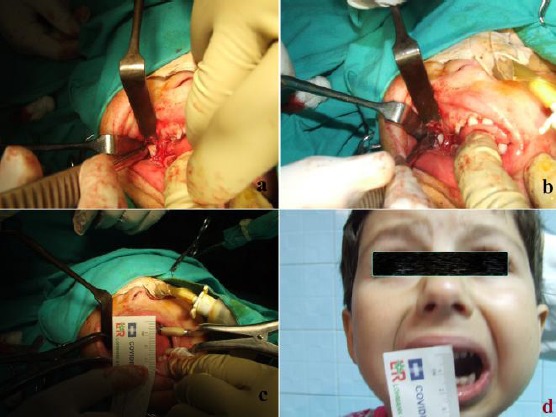
*a. Osteotomy to the right coronoid process of mandible b. Transoral coronoidectomy to the right coronoid process of mandible c. Intraoperatively mouth opening of 30 mm; d. 12 mounts postoperative clinical view with an improved mouth opening*.

The coronoid processes were approached through the incision along with stripping of the temporalis attachment on the anterior border of ramus of mandible and cutting of tendon attachments (Fig. 2).

The first step of osteotomy was made to the right coronoid process of mandible (Fig. 2). After unilateral (right side) coronoidectomy, the second step was followed by ipsilateral (left side) osteotomy and coronoidectomy through the same exposure to remove the coronoid and recording of maximum mouth opening of 30mm (Fig. 2).

Coronoidectomy was done through an intraoral approach followed with mouth opening of 30 mm (satisfactory distance between upper and lower lip of the mouth). Surgical procedure was performed under general anesthesia with nasotracheal intubation, guided by a nasofiber endoscope.

Alginate impressions were taken intra-operatively to fabricate a splint. Postoperatively the parents were advised for intensive daily mandibular physiotherapy with device TheraBite.

One week after surgery, rehabilitation was begun using a TheraBite 3-5 times a day for 5-10 minutes. Two and six weeks postoperatively, muscular stretching and forced mouth opening under general anesthesia were performed.

As a final result, a mouth opening of 25 mm was achieved, enabling the patient to consume a normal diet and take care of her teeth (Fig. 2).

The patient was follow-up postoperatively for at regular intervals after three months, six months, nine months and one year and we found no reduction of mouth opening established with previous treatment.

*Ethical approval:* All procedures performed in studies involving human participants were in accordance with the ethical standards of the institutional and/or national research committee and with the 1964 Helsinki declaration and its later amendments or comparable ethical standards.

## Discussion

Coronoid hyperplasia negatively influences growth and development of the jaws and teeth; because of the facial deformity, which worsens with growth, psychosocial development of the patient is affected as well [5, 6].

Etiopathogenesis of bilateral forms of coronoid hyperplasia has been seen limited and has not yet been described, it should be to both developmental and endocrine abnormalities, followed by a familiar pattern of inheritance.

The poor specificity of signs and symptoms associated with this type of bilateral coronoid hyperplasia present problems of differential diagnosis.

Three-dimensional computed tomography with reconstruction is necessary and fundamental for differentiation of the coronoid hyperplasia from other conditions such: ankilosis, coronoid neoplasms (chondroma or osteochondroma), trauma and thickened but not elongated coronoid processes [7]. This report also emphasizes the significance of three- dimensional CT techniques in diagnosis and surgical planning. The main aim of treatment is to restore the mouth opening, in a stable manner.

Intraoral and extraoral approach are described in literature, with intraoral being more recommended for preventing external scars and facial nerve injury [8]. We performed intraoral approach coronoidectomy; regardless the restricted access to the operatory field, the procedure was successful. Some authors prefer coronar approach to reduce the risk of hematoma formation and avoid intraoral scarring [3, 5].

Intensive postoperative physical therapy is critical for clinical success; treatment failure is usually due to inadequate physical therapy. In this case the device TheraBite was used [9, 10].

We strongly suggest early surgical treatment to recover morphology and function, consequently reducing skeletofacial deformities in young patients.

In conclusion, coronoid process hyperplasia depending on their location and extent can cause mandibular hypomobility. Setting the diagnosis of coronoid hyperplasia as soon as possible and its distinguishing from other forms of ankylosis by using three-dimensional computed tomography is very important. While a considerable level of mouth opening reduction and restricted mandible’s function is present, a coronoidectomy by intraoral approach followed by an intensive physiotherapy (begin between 1-2 weeks after surgery with stretching and passive movements) gives satisfactory results in treatment of this condition.

Coronoid process hyperplasia is an uncommon finding, characterized by an enlargement of the coronoid process, causing a mechanical obstacle by its interposing in the posterior portion of the maxilla or zygomatic arch. Panoramic x-ray and 3-dimensional computed tomographic reconstruction in our manuscript showed bilateral elongation of the coronoid processes associated with deformation of the mandibular condyle with no involvement of the articular space. We strongly suggest early surgical treatment of coronoid hyperplasia to recover morphology and function consequently to reduce skeletofacial deformities in young patients.
